# The Emerging Role of Cold Atmospheric Plasma in Implantology: A Review of the Literature

**DOI:** 10.3390/nano10081505

**Published:** 2020-07-31

**Authors:** Wang Lai Hui, Vittoria Perrotti, Flavia Iaculli, Adriano Piattelli, Alessandro Quaranta

**Affiliations:** 1 Private Practice, Smile Specialists Suite, Newcastle 2300, Australia; drlizzyhui@gmail.com; 2 Department of Medical, Oral and Biotechnological Sciences (DSMOB), University of Chieti-Pescara, 66100 Chieti, Italy; apiattelli@unich.it; 3 Department of Neuroscience and Reproductive and Odontostomatological Sciences, University of Naples Federico II, 80131 Naples, Italy; f.iaculli@unich.it; 4 Sydney Dental Hospital, Sydney 2010, Australia; profalexquaranta@gmail.com; 5 Scientific and Education Director, Smile Specialists Suite, Newcastle 2300, Australia

**Keywords:** biofilm, cold atmospheric plasma, decontamination, wound healing, oncology, dentistry, implantology

## Abstract

In recent years, cold atmospheric plasma (CAP) technologies have received increasing attention in the field of biomedical applications. The aim of this article is to review the currently available literature to provide an overview of the scientific principles of CAP application, its features, functions, and its applications in systemic and oral diseases, with a specific focus on its potential in implantology. In this narrative review, PubMed, Medline, and Scopus databases were searched using key words like “cold atmospheric plasma”, “argon plasma”, “helium plasma”, “air plasma”, “dental implants”, “implantology”, “peri-implantitis”, “decontamination”. In vitro studies demonstrated CAP’s potential to enhance surface colonization and osteoblast activity and to accelerate mineralization, as well as to determine a clean surface with cell growth comparable to the sterile control on both titanium and zirconia surfaces. The effect of CAP on biofilm removal was revealed in comparative studies to the currently available decontamination modalities (laser, air abrasion, and chlorhexidine). The combination of mechanical treatments and CAP resulted in synergistic antimicrobial effects and surface improvement, indicating that it may play a central role in surface “rejuvenation” and offer a novel approach for the treatment of peri-implantitis. It is noteworthy that the CAP conditioning of implant surfaces leads to an improvement in osseointegration in in vivo animal studies. To the best of our knowledge, this is the first review of the literature providing a summary of the current state of the art of this emerging field in implantology and it could represent a point of reference for basic researchers and clinicians interested in approaching and testing new technologies.

## 1. Introduction

In recent years, plasma-enabled biomedical technologies have emerged as a promising approach for non-chemical, low-temperature decontamination in the biomedical, food manufacturing, and food service industries. Their use in medicine extends into synergistic and personalized plasma-enabled therapeutics for tissue regeneration [1,2], oncotherapy [3,4], and dermatology [5]. A rapidly growing body of evidence documents their use in disinfecting living and abiotic targets, promoting cell differentiation and migration, and enhancing tissue regeneration and wound healing. Among the novel applications of non-equilibrium plasma, biomedical applications, such as electrosurgery, surface modification of biocompatible materials, and decontamination of heat-sensitive medical tools, may be particularly interesting for future dental applications [6].

## 2. Overview of Plasma Features and Functions

Plasma, referred to as the fourth state of matter, is an electrically neutral ionized gas that has antibacterial properties through the generation of a mixture of reactive oxygen and nitrogen species (RONS), excited molecules, charged particles, chemically reactive neutral particles, and ultraviolet (UV) radiation [7,8]. The composition of the reactive components in plasma depends on the type of source used, as well as the applied operational conditions and parameters [9].

Plasma can be divided into high-temperature, thermal, and non-thermal groups [10]. In high-temperature plasma, all particles (electrons and heavy particles) have the same temperature, and they are therefore in thermal equilibrium. In thermal (quasi-equilibrium) plasma, there are only areas of thermal equilibrium within the plasma; energy is used to heat the entire gas, and temperatures often range from 10,000 to 100,000 K (1–10 electron volts (eV)), limiting its applicable use; moreover, it presents both electrons and heavy particles (neutrals and ions) at the same temperature. Finally, non-thermal (non-equilibrium) plasma has particles that are not in thermal equilibrium. This plasma is termed “cold atmospheric plasma” (CAP) [10]; it has only heavy particles at room temperature, resulting in a point of application of ≤ 40 °C [11]. CAP can be obtained by various gases, such as helium, argon, nitrogen, heliox (a mixture of helium and oxygen), and air [11]. The clinically used and experimentally tested CAP devices can be divided into three main categories: (1) those based on direct discharge (DBD); (2) those based on indirect discharge, and (3) hybrid types [2]. The DBD devices provide a higher intensity and more adaptable and controlled discharge. They can also generate plasma solely in air without the need for carrier gases. Indirect discharge is generated by devices usually called plasma jets, plasma pens, or plasma torches. The hybrid plasma devices are currently applied only at the experimental level [2]. Depending on its settings, each device would have several effects on biomedical applications [12]. Considering the low temperature of application, as well as the acceptable thermal damage to tissues [13,14,15,16], CAP has been suggested as a promising device, not only for systemic biomedical treatments, but also as a chairside approach [2,17] for the treatment of different oral diseases.

UV radiation, ions, and electrons produced by plasma play an important role in enhancing its physical effectiveness, and free radicals provide strong oxidative effects on the outer structures of cells [18]. Indeed, charged particles and RONS produced by plasma are able to greatly compromise the integrity of the walls, coats, and membranes of bacterial cells [6,19,20], resulting in microbe inactivation [21]. When directly applied to tissues or cells, plasma treatment can alter cellular activities in both prokaryotes and eukaryotes, and thus control and manipulate the biological processes fundamental to biofilm formation, tissue regeneration, and carcinogenesis [3]. Moreover, plasma treatment could result in increased cell proliferation, cell spreading, and the synthesis of the proteins of the extracellular matrix [22], as previously demonstrated by osteoblast-like cells that were able to create highly advanced cellular networks when cultured on CAP-conditioned titanium surfaces [23,24,25]. However, it should be taken into account that the antimicrobial effect, as well as the stimulus of cell proliferation, are not obtained with the same application of the same plasma source. Indeed, further studies are needed for a precise definition of CAP conditions of use and parameters for a specific biological target in order to obtain the desired effect. More intense treatment may also be customized for surface decontamination, whereby plasma etching can remove biomolecules, such as proteins, pyrogens, or extracellular polymeric substances [26].

In addition, appropriate plasma applications may improve a surface’s wettability and modify the oxide layer that, in turn, interacts with the proteins and cells of surrounding tissues, thus enhancing tissue and cell adhesion [7,27,28,29,30]. It should be noted that an increase in surface hydrophilicity is not necessarily related to surface damage or to the enhancement of surface roughness [17], although this aspect is still under debate.

## 3. Plasma’s Potential Biomedical Applications

CAP has showed encouraging results in decontamination, blood clotting, skin disease treatment, cancer therapy, and oral medicine [12].

### 3.1. Systemic Applications

In medicine, CAP treatment may be successfully applied in dermatology [5], blood coagulation [31], surgical instrument and consumable decontamination [32], and the hydrophilic property enhancement of the surfaces of biomaterials [27]. Due to its antimicrobial effects, CAP has also been proposed for water disinfection since its application determines a series of exposure and postexposure channel reactions, which result in water purification [33].

The potential use of CAP in clinical oncology has been recently analyzed and it is obtaining a growing interest in the scientific community [11]. As currently reported by Semmler et al. [4], plasma application would induce tumor cell death (i.e., necrosis, apoptosis, senescence, and autophagy) in a dose-dependent manner, as well as decrease their adhesion, migration, and invasion, reducing cancer cell diffusion and metastasis forming ability [4]. Specifically, the same authors reported promising results in the treatment of head and neck squamous cell carcinomas in terms of lesion regression as well as pain reduction. However, the underlying mechanism determining the tumor cell arrest and the relative immune response have not been elucidated yet and need further evaluation.

Another promising field for CAP application is dermatology [5], as demonstrated by the absence of both damage of the skin barrier and a reduction in skin hydration following plasma usage [13]. Moreover, when applied in vivo, plasma treatment may speed up tissue granulation and enhance wound healing [14,34,35]; Daeschlein et al. [36] showed the ability of CAP to restrain the microbial colonization of chronic wounds. Although promising, the dermatological application of CAP should be studied in depth to render the treatments more effective and stable over time.

### 3.2. Oral Applications

In the oral medicine field, CAP has been applied to the treatment of dental caries, periodontal disease, implantology, teeth whitening, endodontic infection, tooth remineralization, an increase in the bonding efficacy of composite resin, and the disinfection of dental instruments [11,12,17,37]. The idea of using CAP for innovative dental procedures was first proposed by Goree et al. [38], who demonstrated the ability of a plasma needle to reproducibly kill the most cariogenic bacterium, *Streptococcus mutans*, thus attracting the interests of researchers from the dental field.

Since then, the largest area of investigation into CAP has dealt with endodontics. Indeed, the elimination of bacteria in infected root canals, especially with persistent periapical lesions, still remains an unsolved issue, as conventional chemical irrigants fail to achieve the eradication of bacteria in the root canals [39]. For these reasons, CAP can be seen not only as an alternative but also as an adjunct to investigate synergistic treatments. An interesting study comparing the antimicrobial efficiency of plasma jets with chemical irrigation solutions, such as chlorhexidine (CHX) and Sodium hypochlorite (NaOCl) against the principal organism responsible for endodontic treatment failures, *Enterococcus faecalis*, was conducted in a standardized simulated root canal model [39]. The results of this in vitro study showed that the plasma treatment achieved a significantly higher microbial reduction than chemical 0.1% CHX irrigation (*p* < 0.001) and a comparable one with 0.6% NaOCl. However, the conditions used in this study were far from a “real life” situation. A step forward in that direction was done by Simoncelli et al. [40], who investigated two different procedures for the inactivation of bacteria in realistic tooth models, resembling procedures conventionally adopted in endodontic practice, and using wet and dry canal models. They suggested the possibility of combining direct and indirect treatments in an innovative multi-phase endodontic plasma-based procedure with increased overall antibacterial efficacy. Nevertheless, technical difficulties related to the length of penetration of the plasma plume are hampering its clinical application. Schaudinn et al. [41] used disinfected root canals of extracted teeth to study the effect of non-thermal plasma on ex vivo biofilm and they found an efficacy of biofilm removal lower than that achieved by the traditional treatment of 6% NaOCl, probably due to CAP’s inability to act on bacteria over a longer distance. Therefore, the authors advocated for progress in the development of devices equipped with fine, flexible needles that will ease the disinfection of root canals along their whole length in clinical practice, and future studies aimed at assessing plasma’s effect on the integrity of the treated dental tissue are needed for CAP to reach the dental chair.

#### 3.2.1. Implantology

An electronic search was conducted through PubMed, Medline, and Scopus databases to identify articles dealing with the use of CAP in implantology. The following keywords were used alone and in combination: “cold atmospheric plasma”, “argon plasma”, “helium plasma”, “air plasma” “dental implants”, “implantology”, “peri-implantitis”, “decontamination”. The search resulted in 23 papers [7,12,18,21,22,27,28,32,37,42,43,44,45,46,47,48,49,50,51,52,53,54,55] which aimed at investigating the effects of CAP on biocompatibility, surface improvement, and cleaning efficacy (Table 1). Only five [22,45,46,47,49] out the 23 studies were in vivo studies on animal models.

The antimicrobial and surface modification plasma potential demonstrated in in vitro and in vivo models would suggest CAP as a promising option in the treatment of peri-implantitis [30,56], although further evidence is necessary to draw final conclusions. CAP may enhance the elimination of bacterial plaque from implant surfaces, in inaccessible pockets or during open-flap debridement, and should stimulate the process of the re-osseointegration of affected dental implants [57] by enhancing their wettability. Indeed, the potential to determine a super-hydrophilic surface may stabilize the blot clot and promote the early wound healing immediately after implant insertion [17,23]. To better analyze the encouraging application of CAP in implantology, its effects were divided by biocompatibility property, surface improvement, and antimicrobial activity.

##### Biocompatibility

The influence of the CAP treatment of titanium and zirconium discs on cell activities has been investigated in a few in vitro studies [7,12,27,28,48,52,54], and they generally agree on its supportive role in cell adhesion, spreading, and proliferation. Specifically, when the treatment was conducted on titanium surfaces, Duske et al. [7] reported that the size of osteoblastic cells grown on argon–oxygen plasma-treated titanium discs was significantly larger than on non-treated surfaces irrespective of surface topography (machined, sandblasted and acid-etched SLA, SLActive, subjected to airflow or diamond bur application). Accordingly, higher osteoblastic cell adhesion and positive cell morphology were reported on plasma-conditioned titanium surfaces than untreated surfaces [43,44]. Moreover, the combination of CAP treatment with the mechanical brushing of titanium samples seemed to determine a clean surface with cell growth comparable to the sterile control [48]. Enhanced osteoblastic cell proliferation and viability have also been shown on zirconia surfaces treated with oxygen CAP, providing significantly better results than in the same cells cultured on nontreated, UV-treated, and argon plasma-treated specimens [54]. In the same way, CAP appeared to improve fibroblast cell colonization and adhesion both on titanium and zirconia surfaces [28,42], mainly in the early phase of culture.

Besides, Tominami et al. [58] observed the effect of CAP irradiation on culture media containing plated pre-osteoblastic MC3T3-E1 cells, concluding that an accelerative effect on cell mineralization due to alkaline phosphate (ALP) activity enhancement and osteoblastic differentiation improvement could be suggested.

##### Surface Improvement

The potential of CAP to change the physico-chemical properties of the titanium surfaces, without affecting their microstructure [32], may play a central role in surface “rejuvenation” that, in turn, promotes the re-osseointegration of previously affected dental implants [59]. The enhancement of wettability could be considered as a promising tool in the treatment of peri-implantitis [48], inducing not only an improvement in osteoblast as well fibroblast cells spreading, but also increasing the immune cells essential to eliminating residual bacteria [48,60].

CAP application has demonstrated its efficacy to reduce the in vitro water contact angle (WCA) of treated titanium surfaces [7,28,42,52], resulting in an improvement of hydrophilic surface features. Yang et al. [12] even demonstrated an improvement in surface roughness after plasma treatment, that may contribute to the enhancement of subsequent cell adhesion. As demonstrated by a recent in vivo study [22], CAP conditioning of sand-blasted and acid-etched titanium dental implants prior to implant placement resulted in the absence of morphological surface alterations, as well as an improvement in osseointegration parameters (expressed as bone-to-implant contact—BIC) after 8 weeks of healing. Histological analysis provided by the same study [22] showed the homogenous mineralization of newly formed bone, suggesting a promising use of CAP therapy before dental implant positioning.

Zirconia has been demonstrated to positively respond to CAP treatment, showing an absence of structure alterations and surface oxidation and an increase in wettability following oxygen plasma or argon plasma applications for 12 min [54]. In addition, helium CAP treatment on zirconia discs only demonstrated a change in surface chemistry but not in surface topography, suggesting a promising role in the decontamination of zirconia abutment, as well as an improvement in soft peri-implant tissues, which may prevent peri-implant lesions over time [21].

##### Antimicrobial Activity

The effect of CAP in surface decontamination, as well as antimicrobial efficacy, may indicate plasma as a suitable device in the treatment of peri-implantitis. The presence of plaque as an etiological factor of peri-implant lesions [61] stresses the need for a therapy with a reliable cleaning efficacy, even in an anatomically disadvantaged situation. In this regard, Pei et al. [62] assessed the CAP depth of penetration using a mobile (wireless) handheld plasma device to inactivate an *Enterococcus faecalis* biofilm of 25.5 µm in thickness, which is essentially 17 layers of cells. The authors demonstrated effective penetration of the plasma-generated reactive oxygen species to the very bottom layer after 5 min of treatment at a 5 mm distance.

Argon plasma has demonstrated, in cell culture, a significant reduction of *Streptococcus sanguinis* biofilms [63] and the ability to disinfect titanium discs contaminated with *Aggregatibacter actinomycetemcomitans* [57]. Accordingly, helium plasma showed a bactericidal effect against *Porphyromonas gingivalis* biofilms grown on sandblasted, large grit, acid-etched (SLA) discs, mainly when applied for more than 3 min [37], as well as a bacterial growth inhibition and a decrease in biofilms of *Streptococcus mutans* and *Porphyromonas gingivalis* on zirconia specimens [21].

When compared to different titanium implant decontamination methods, such as laser radiation with diode devices, air abrasion, and chlorhexidine (CHX), the exposure of titanium machined discs to CAP significantly reduced the viability and quantity of oral biofilms compared with the other tested treatments, although a complete biofilm removal was not obtained [18]. Accordingly, three different plasma devices were more effective in removing multispecies human saliva biofilms grown on titanium discs, when compared to CHX application [64]. Preissner et al. [53] compared the in vitro effect of CAP, for both 60 and 120 s, to that of diode laser irradiation for 60 s on *Streptococcus mitis* biofilms cultivated on microrough titanium dental implants. Both type of CAP treatments resulted in a greater reduction of adhering bacteria than laser application [53]. Similar results were reported by Ulu et al. [55], who compared contact and non-contact Er:YAG (erbium-doped yttrium aluminium garnet) lasers with CAP used on SLA discs contaminated by *Staphylococcus aureus* biofilms. CAP not only performed better than the evaluated lasers, but may also be used in a safer way since the potential thermal damage to the bone and surrounding tissue was mainly caused by the contact laser that showed a focal temperature increase of up to 58 °C [55].

The treatment of peri-implant lesions was demonstrated to be more effective when different surgical or non-surgical approaches were combined [65,66,67]. In this view, Shi et al. [68] showed that the combination of the conventional techniques (e.g., the elevated flaps, curetted plaque, calculus, and granulation tissue, irrigated with 0.2% CHX digluconate and sterile saline solution) and CAP could lead to a higher bone level, a significantly decreased detection of bacteria (*Porphyromonas gingivalis* and *Tannerella forsythia*), and to a significant improvement in the clinical examination. Precisely, the association of mechanical treatments (such as mechanical brushing or air polishing) and CAP [23,40,44] have already provided promising results for dental implant decontamination, highlighting the synergistic antimicrobial effect and surface improvement that may represent an encouraging method in the treatment of implants affected by peri-implantitis.

##### Results from In Vivo Studies

To the best of our knowledge, only five studies [22,45,46,47,49] compared the differences in the osseointegration of untreated as well as CAP-conditioned rough titanium [22,46,47] and calcium phosphate-coated (CaP) [47,49] or zirconium implants [45] placed in vivo in animal models (Table 2). The assessment of osseointegration has been conducted on bone biopsies retrieved at different time points (1, 3, 6, and 8 weeks, and 1 or 2 months) and histomorphometrically analyzed. All studies agreed that CAP is a promising option to hasten osseointegration; indeed, significantly higher bone formation was found in Ar CAP treated rough and CaP-coated implants at 3 [45,49] and 8 weeks [22] and in zirconia (ZirTi) at 2 months [38], whilst less evident differences were detected using CAP with the same air composition as the regular atmospheric composition (16% oxygen, 1% hydrogen, and 78% nitrogen). None of the studies above investigated the mechanism underneath the improvement of the quantity and quality of bone healing. Only Naujokat et al. [22] tried to gain information about the chronological sequence of bone formation by labeling bone metabolism, but they did not find a relevant discrepancy of fluorescence between untreated and CAP-treated implants. Therefore, it can be concluded that CAP’s beneficial effect on osseointegration and “reosseointegration” is worthy of further investigation in prospective clinical trials.

#### 3.2.2. Future Trends in Oral Surgery and Implantology

In addition to the highly efficient removal of biological residuals from implant surfaces [21,37,53,55,57,62,63,64], plasma treatment can also be an effective tool for lasting and highly controlled surface modification [69], including, but not limited to, chemical functionalization, deposition of antibacterial thin films and coatings [70,71], and surface structuring to create antifouling surfaces. Plasma can also be used for the deposition of highly complex, ordered surface nanostructures from a range of material sources [72], which can exhibit a higher level of control over the attachment behavior of cells and micro-organisms, providing a more selective control tool. Furthermore, plasma deposition has not been limited to dental implants only but may have significant potential in 3D porous scaffolds as well. It has been successfully shown to impart chemical gradients inside porous structures to enhance cell viability in comparison to untreated materials [73,74].

The capacity of activating liquids as carriers of antibacterial reactive agents via plasma use may provide a significant advantage in overcoming the limitations related to a lack of direct access to certain areas of the implant surfaces. In fact, with the application of plasma in dentistry (plasma stomatology), the saliva may have a role in the decontamination process. Although, to the best of our knowledge, there is still no research published on the plasma–saliva interactions, the studies on the plasma–liquid interactions, which are among the emerging research lines in the field of plasma science and technology, would promote further specific studies about the abovementioned interaction, which is of special interest for clinical practical applications in the field of plasma stomatology.

## Figures and Tables

**Table 1:** Characteristics of the studies dealing with the use of cold atmospheric plasma (CAP) in implantology.

Authors Year [Reference]	Ti Component/Surface Texture/Implant company	Contamination Method	Number of Specimens Per Group (Total)	CAP Device	CAP Settings: Time (s) Mean Power (W) Gas Distance (D)	Decontamination Methods	Settings for other Methods	Outcome Measured	Overall Conclusions
Rupf et al. 2011 [32]	- Ti discs; - Sandblasted acid-etched; - Friadent, Mannheim, Germany	Oral biofilm formed in situ by fixing Ti at the buccal site of molar and premolar teeth for 24 h or 72 h	149:24 h 149:72 h 36: no biofilm (334)	Custom built (Leibniz Institute of Surface Modification, Germany)	- 3 or 5 W; - He 2.0 slm; - D: 2 mm	- No treatment; - CAP 3W; - CAP 5W; - Air/water spray; - CAP 3W + air/water spray; - CAP 5W + air/water spray; - CAP 3W + air/water spray + CAP 3W; - CAP 5W + air/water spray + CAP 5W	- 2 bar; - 5 s; - D: 10 mm	- Biofilm thickness; - Biofilm viability; - Biofilm vitality; - Total protein	CAP caused inactivation of bacteria biofilm and significant reduction of protein amounts. For complete elimination, additional application and second series of CAP was necessary
Coelho et al. 2012 [46]	- Root form Ti implants; - (AB/AE); - Integra-Ti, Bicon LLC, Boston)	No contamination	24 implants	kiNPen (INP, Greifswald, Germany)	- 60 s per quadrant; - Ar	n/a	n/a	- SE; - Surface characterization; - Surface chemical assessment; - BIC; - BAFO	CAP fostered higher levels of contact with surrounding tissues, promoting more rapid ad higher quantity of bone around rough Ti surfaces
Duske et al. 2012 [7]	- Ti discs; - Machined/coarse diamond grit/airflow-treated/sandblasted etched; - SLActive, Straumann, Freiburg, Germany	No contamination	10 discs per group (360)	Plasma jet (INP, Greifswald, Germany)	- 30 and 60 and 120 s; - 2-3 W; - Ar/Ar and O_2_ 1%/Ar and O_2_ 0.2% 5 slm; - D: 5 mm	- 1a. Ar CAP 30 s; 1b. Ar CAP 60 s; - 1c. Ar CAP 120 s; - 2a. 0.2% O_2_ Ar CAP 30 s; - 2b. 0.2% O_2_ Ar CAP 60 s - 2c. 0.2% O_2_ Ar CAP 120 s - 3a. 1.0% O_2_ Ar CAP 30 s - 3b. 1. 0% O_2_ Ar CAP 60 s - 3c. 1.0% O_2_ Ar CAP 120 s	n/a	- Disc topography; - Contact angle measurement; - MG-63 area, morphology, metabolic activity	CAP reduced contact angle and supports spreading of MG-63 cells
Canullo et al.2013 [42]	- Ti discs; - Smooth; - Sweden & Martina	n/a	30 per group (60)	Plasma Reactor (Colibri, Gambetti Company)	- 360 s - 10 W	Untreated	n/a	L 929 viability, adhesion, morphology	CAP treatment could be used for abutment cleansing to favor peri-implant tissue healing
Giro et al. 2013 [49]	- Root form Ti implants; - CaP; - Integra-CP, Bicon LLC, Boston	No contamination	24 implants	kiNPen (INP, Greifswald, Germany)	- 20 s per quadrant; - Ar 4.0 slm	n/a	n/a	- Surface energy; - Surface chemical assessment; - BIC; - BAFO	Higher degrees of surface wettability resulted in significantly higher BIC and BAFO following CaP-CAP
Idlibi et al. 2013 [18]	- Ti discs; - Machined; - Friadent, Mannheim, Germany	Oral biofilm formed in situ at the buccal site of molar and premolar teeth for 72h	20 in each group (200)	Custom built (Leibniz Institute of Surface Modification, Leipzig, Germany)	CAP 1: - 196.25 s - 5 W; - He 2.0 slm; - D: 2 mm CAP 2: - 196.25 s; - 3W; - He 2.0 slm; - D: 2 mm CAP 3: - 49.06 s; - 5W; - He 2.0 slm; - D: 2 mm CAP 4: - 49.06 s; - 5W; - He 2.0 slm and O_2_ sccn; - D: 2 mm	1. Untreated control; 2. Gas; 3. DL; 4. AA; 5a and 5b. CHX	2. - 196.25 s; - He 2.0slm; - D: 2mm 3. - 2.5 W; - 25 ms pulse and 50 ms pause. 4. - Amino acid glycine powder; - 5a. - 60 s - 5b. - 200 s	- Biofilm viability; - Biofilm quantity; - Biofilm morphology	CAP significantly reduced the viability and quantity of biofilm, although complete removal was not achieved. Its efficacy correlated with the treatment duration and CAP power
Danna et al. 2015 [47]	- Root form Ti implants; - GB/AE and CaP; - Integra-Ti, Bison LLC, Boston, MA	No contamination	56 implants	kiNPen (INP, Greifswald, Germany)	- 20 s per quadrant; - 16% O_2_, 1% H, 78% N 5 slm	n/a	n/a	- Surface physical characterization; - Surface morphology; - SE; - Surface chemical characterization; - BIC; - BAFO	CAP-treated Ti and CaP implants showed decreased levels of C and increased levels of Ti and O, Ca and O. No significant differences for BAFO. Significant increase in BIC for CAP-treated Ti implants, not for CaP surfaces
Duske et al. 2015 [48]	- Ti discs; - Sandblasted etched; - Straumann, Freiburg, Germany	Biofilm formed in vitro from subgingival plaque	10 discs per group	kINPen08, INP Greifswald, Germany	- 540 s per disc (60 s per spot); - 2–3 W; - Ar 99%, O_2_ 1% 5 slm; - D: 5 mm	Untreated BR BR+CAP 4. Auto	BR 1 mm/s for 120 s	- Biofilm morphology; - MG-63 cell morphology, area and number; - Biofilm regrowth; - Cell growth	Biofilm remnants on BR and CAP impaired MG-63 cell development, whilst BR+CAP provided an increased area of MG-63 cells
Ibis F et al. 2016 [50]	- Ti discs; - 304 SS, 316 SS, Ti6Al4V, UHMWPE; - Hipokrat Medical Devices A.S., Izmir Turkey	*Escherichia coli*; *Staphylococcusaureus*	n/a	Custom made	n/a	n/a	n/a	- Contact angle measurement; - Biofilm viability	Up to > 95% biofilm was inactivated by CAP and up to 50% was retarded. Increased hydrophilicity after CAP was obtained.
Lee et al. 2016 [51]	- Ti discs; - Machined/ASD/RBM/sandblasting SLA; - N/A	No contamination	n/a	Custom made	Pure He/He and O_2_D: 20 mm	n/a	n/a	- Optical emission; - Wettability	CAP treatment enhances wettability of the Ti surfaces especially for the He/O_2_ CAP
Preissner et al. 2016 [53]	- Ti implants; - Sandblasted acid-etched micro-rough surface + 0.5 machined collar; - Tiny implant, BTI Biotechnology Institute, Minano, Spain	Streptococcus mitis	Eight implants per group (32)	TTP60 and TTP120, kINPen Med (INP Greifswald, Germany)	TTP 60: 60 s; Ar 4.3 slm;	Negative control (1% sodium chloride) DL GaAlAs	1. - 60 s; 2. - 60 s at 2.0 W	- Bacterium identification; - Bacterial adhesion	Number of dead cells was higher with CAP compared to DL and control
					TTP 120: 120 s; Ar 4.3 slm				n/a
Canullo et al. 2017 [44]	- Ti discs - Machined/plasma sprayed/zirconia-blasted; - Sweden & Martina	Streptococcus mitis	(720)	Plasma beam mini (Diener Electronic)	- 120 s - 8 W - D: 2mm	n/a	n/a	- Bacterial adhesion; - MC3T3-E1 adhesion, morphology and viability; - Contact angle measurements; - Protein adsorption	CAP enhanced MC3T3-E1 attachment and spreading as well as bacterial decontamination
Canullo et al. 2017 [43]	- Ti discs - Machined/plasma sprayed/zirconia-blasted and acid-etched; - Sweden & Martina	No contamination	92 discs per group (216)	Plasma R (Sweden & Martina)	- 720 s - 10 W	Untreated	n/a	- Surface morphology; - Contact angle measurements; - MG-63 morphology, adhesion	CAP showed a positive effect on MG-63 cells grown on CAP-treated and untreated machined, plasma sprayed, and zirconia discs.
Matthes et al. 2017 [52]	- Ti discs - Sandblasted acid-etched; - Biomet 3i LLC, Palm Beach Garden, FL, USA	Biofilm formed in vitro from subgingival plaque	200	kINPen09, neoplas GmbH, INP Greifswald, Germany	- 300 or 720 s; - 3.5 W; - Ar 5 slm; - D: 5mm	AA AA + CAP	1 and 2 Erythritol for 90 s	- MG-63 adhesion and morphology; - Water contact angle	AA + CAP did not enhance MG-63 spreading compared to AA alone.
Matthes et al. 2017 [52]	- Ti discs - Sandblasted acid-etched; - Biomet 3i LLC, Palm Beach Garden, FL, USA	Biofilm formed in vitro from subgingival plaque	200	kINPen09, neoplas GmbH, INP Greifswald, Germany	- 300 or 720 s; - 3.5 W; - Ar 5 slm; - D: 5mm	AA AA + CAP	1 and 2 Erythritol for 90 s	- MG-63 adhesion and morphology; - Water contact angle	AA+CAP did not enhance MG-63 spreading compared to AA alone.
Karaman et al. 2018 [27]	- Ti discs; - RGD (arginine, glycine, aspartic acid) coated; - Titania Medical Devices; Izmir, Turkey	No contamination	n/a	Custom made	n/a	RGD RGD + CAP	n/a	- Surface characterization; - Contact angle measurements; - Surface topography; - hMSC attachment, morphology, and proliferation	RGD + CAP significantly increased cell adhesion and proliferation
Canullo et al.2018 [45]	- Implants - ZirTi surface - Premium Sweden & Martina, Due Carrare, Italy	No contamination	Four implants per animal (eight beagle dogs)	Ar-plasma (Diener electronic, Jettingen, Germany)	- 720 s - 75W	Untreated	n/a	- Old bone - New bone - Total mineralized bone - Soft tissues	Implants treated using AR-plasma reached higher BIC when compared to untreated
Ulu et al. 2018 [55]	- Ti discs; - SLA; - NucleOSS, Izmir, Turkey	*S. aureus*	76	Plasma One (Plasma Medical Systems, Bad Ems, Germany)	- 120 sec - 5 W; - D: 1 mm	Laser ER:YAG	30 s at 100mJ/pulse power	- Antimicrobial activity; - Biofilm viability; - Surface roughness	Cap showed superior antibiofilm activity than contact and noncontact laser treatment without temperature increase or damages to the surface of Ti discs
Yang et al. 2018 [12]	- Ti A_2_; - N/A; - Northwest Institute for Non-Ferrous Metal Research. Xi’an, China	*Porphyromonas gingivalis*	n/a	Custom made	- CAP1: 120 s - CAP2: 240 s - CAP 3: 360 s - D: 1.5cm	Untreated	n/a	- Surface chemical characterization; - Surface roughness measurement; - Water contact angle measurement; - Bacterial morphology; - MG-63 and MC3T3-E1 growth rate	CAP improved surface hydrophilicity and roughness and completely eliminated *P. ginigvalis* in 360 s, promoting growth of both cell lines
Lee et al. 2019 [37]	- Ti discs; - Sandblasted etched Ti discs; - Osstem Implant Co., Ltd., Busan, Korea	*P. gingivalis*	Five discs per group, two discs per group	Custom made	- He 5 slm; - D: 30 mm	UntreatedHe without CAPHe + CAP	n/a	- Bacterial count; - Bacterial viability; - Bacterial morphology	He-CAP was effective for removing *P. gingivalis* from SLA discs without surface alterations
Matthes et al. 2019 [28]	- Zirconia discs; - Yttria-stabilized zirconium dioxide, polished and sintered; - VITA Zahnfabrik H. Rauter	No contamination	n/a	kINPen09, kINPen08 and kiNPen Chamber, (INP Greifswald, Germany)	- kiNPen 09 e kiNPen 08: - 300 s per side; - Ar 5.5 slm; - D: 5 mm	0.2% CHX;0.1% octenidine;70% ethanol	Antiseptic solutions for 900 s	- HGF-1 cell area, adhesion and morphology; - Water contact angle	CAP reduced water contact angle and supported cell coverage, whereas CHX and octenidine reduced cell surface coverage.
					- Chamber - 300 s per side; - Ar 5 slm - D: 10mm				
	- Ti discs; - Smooth; - Sirona Dental Systems								
Naujokat et al. 2019 [22]	- Ti implants; - Abrasive-blasted, acid-etched, and calcium phosphate-coated; - Camlog, Screw Line, Camlog Biotechnologies AG, Basel, Switzerland	No contamination	16 implants	kINPen Med, INP Greifswald, Germany	- 240 s, - Ar 4.3–4.4 slm; - D: 7 mm;	Untreated	n/a	- Surface morphology; - BIC; - ITBD; - PIBD	CAP did not lead to remarkable change in surface morphology. CAP conditioning prior to insertion resulted in higher BIC and ITBD, but not faster or stronger bone adherence and mineralization
Smeets et al. 2019 [54]	- Zirconia discs; - Yttria-stabilized zirconia containing 5% yttria; - Moje Keramik-Implantate	No contamination	(364)	Yocto III (Diener Electronic)	CAP 1 - 720 s; - O_2_ CAP 2 - 720 s; - Ar	1. UV	1a. - λ 250 nm; - 2mW/cm^2^ 1b. - λ 360 nm; - 0.05 mW/cm^2^	- Surface structure, topography; - Surface wettability; - Surface chemistry; - MC3T3-E1 attachment, morphology, viability, and proliferation	CAP and UV caused a significant reduction of organic material, increased the hydrophilicity of zirconia, and improved the conditions for osteoblasts
	- Ti discs; - Machined; - Camlog, Biotechnologies AG, Basel, Switzerland								
	- Polyurethane; - RM-A; - Hatano Research Institute								
Yang et al. 2020 [21]	- Zirconia discs; - Yttrium-stabilized; - Wieland, Pforzheim, Germany	*S. mutans*; *P. gingivalis*	n/a	CAP Med-I (Plasma Health Scientech Group, Tsinghua University, China)	CAP1 - 30 s; - He 13.5 slpm - D: 1 cm CAP2 - 60 s - He 13.5 slpm - D: 1 cm CAP2 - 90 s - He 13.5 slpm - D: 1 cm	Untreated	n/a	- Surface topography; - Surface wettability; - Surface chemical composition; - Bacterial adhesion, morphology, viability; - Biofilm quantification	The He-CAP jet increased hydrophilicity without changing surface topography and eliminated bacterial growth with surface chemistry change.

Abbreviations: Titanium (Ti); Cold atmospheric plasma (CAP); Alumina-blasted/acid-etched (AB/AE); Surface energy (SE); Bone-to-implant contact (BIC); Bone area fraction occupancy (BAFO); Human osteoblastic cells (MG-63); Calcium phosphate (CaP); Argon (Ar); Helium (He); Oxygen (O); Diode laser (DL); Air abrasion (AA); Grit-blasted/acid-etched (GB/AE); Brushing (BR); Autoclaved biofilm (Auto); Anodic spark deposition (ASD); Resorbable blast media (RBM); Sandblasting with large grit followed by acid-etching (SLA); Human mesenchymal stem cells (hMSCs); chlorhexidine (CHX); Interthread bone density (ITBD); Peri-implant bone density (PIBD); Murine fibroblastic cells (L929).

**Table 2:** Results of in vivo studies on CAP-conditioned surfaces implanted in animal models.

Author Year [Reference]	Outcome Measure (Measurement)	Comparison Factor	Results	Conclusions
Coelho et al. 2012 [46]	Histology - BIC - BAFO	AB/AE vs. AB/AE + CAP	*Week 1:* - BIC: AB/AE = AB/AE + CAP - BAFO AB/AE = AB/AE + CAP *Week 3:* - BIC > 300%* - BAFO > 30%	Ar CAP treatment in vivo fostered higher levels of contact with the surrounding tissues and it is a promising option to hasten osseointegration
Giro et al. 2013 [49]	Histology - BIC - BAFO	CaP vs. CaP + CAP	*Week 1:* - BIC: CaP = CaP + CAP - BAFO: CaP = CaP + CAP *Week 3:* - BIC: CaP + CAP > 100% * - BAFO: CaP + CAP > 82% *	Ar CAP-conditioned surfaces supported in vivo a more uniform presence of osteogenic tissue and a closer interaction with the implant surface which may lead to faster and greater osseointegration
Danna et al. 2015 [47]	Histology - BIC - BAFO	- GB/AE vs. GB/AE + CAP - CaP vs. CaP + CAP	*Week 3* - BIC = - BAFO = *Week 6* - BIC: GB/AE + CAP > GB/AE * - CaP + CAP = CaP - BAFO =	Air-based CAP may improve osseointegration of Ti surfaces but not CaP surfaces
Canullo et al. 2018 [45]	Histology - OB - NB - TMB - ST	ZirTi vs. ZirTi + CAP	*1 month* - OB: ZirTi+CAP > ZirTI NB: ZirTi+CAP > ZirTI - TMB: ZirTi+CAP > ZirTI - ST: ZirTi+CAP < ZirTI *2 months* - OB: ZirTi+CAP > ZirTI - NB: ZirTi+CAP > ZirTI * - TMB: ZirTi+CAP > ZirTI * - ST: ZirTi+CAP < ZirTI *	Activation of the implant surface by Ar CAP may enhance the osseointegration process.
Naujokat et al. 2019 [22]	Histology: - BIC - ITBD - PIBD Fluorescence labeling	AB/AE vs. AB/AE + CAP	*Week 8* - BIC: AB/AE + CAP > AB/AE - ITBD: AB/AE + CAP > AB/AE * - PIBD: AB/AE + CAP = AB/AE - Fluorescence: AB/AE + CAP = AB/AE	Ar CAP conditioning resulted in a higher BIC ratio and ITBD, indicating a beneficial effect although neither faster or stronger bone adherence or mineralization was detected by fluorescence labeling

Abbreviations: Bone-to-implant contact (BIC); Bone area fraction occupancy (BAFO); Alumina-blasted/acid-etched (AB/AE); Calcium phosphate (CaP); Grit blasted/acid etched (GB/AE); Old bone (OB); New bone (NB); Total mineralized bone (TMB); Soft tissues (ST); Interthread bone density (ITBD); Peri-implant bone density (PIBD); Cold atmospheric plasma (CAP); Argon (Ar); * Statistical significance.
